# Two cases of granuloma mimicking local recurrence after pulmonary segmentectomy

**DOI:** 10.1186/s13019-020-1055-z

**Published:** 2020-01-08

**Authors:** Mikio Okazaki, Yoshifumi Sano, Yu Mori, Nobuhiko Sakao, Syungo Yukumi, Hisayuki Shigematsu, Hironori Izutani

**Affiliations:** 1 Department of Cardiovascular and Thoracic Surgery, Ehime University Medical School, Toon City, Japan; 2 Department of Surgery, National Hospital Organization Ehime Medical Center, Toon City, Japan

**Keywords:** Lung cancer, Granuloma, Segmentectomy, Polyglycolic acid sheet

## Abstract

**Background:**

Fluorodeoxyglucose (FDG)-positron emission tomography (PET)/CT is the most sensitive non-invasive imaging method for the detection of tumor metastasis and recurrence, but sometimes reveals false-positive results. Herein, we report two cases of false-positive results on PET/CT scans along with elevated serum carcinoembryonic antigen (CEA) levels, mimicking local recurrence after pulmonary segmentectomy.

**Case presentation:**

Case 1; A 75-year-old woman underwent thoracoscopic left basal segmentectomy for primary lung cancer. Follow-up at 6 months after the surgery revealed serum CEA level elevation and chest CT showed a nodule measuring 25 × 22 mm in the residual left lower lobe. PET/CT revealed FDG uptake in the nodule diagnosed as local recurrence of lung cancer, and the patient underwent partial resection of the nodule. Microscopic examination of the resected specimen revealed granuloma caused by polyglycolic acid (PGA) sheet. Case 2; A 58-year-old man underwent VATS right S1 segmentectomy for lung metastasis from rectal carcinoma. Serum CEA levels gradually increased after surgery, and PET/CT revealed FDG uptake in the stump diagnosed as local recurrence of the lung metastasis. The patient underwent completion lobectomy 6 months after the segmentectomy, and the pathology of the resected specimen revealed an inflammatory granuloma caused by PGA suture.

**Conclusions:**

Although suture and stapler granulomas have been reported, granuloma caused by PGA sheets has never been reported. Postoperative recurrence of lung cancer should be diagnosed with not only PET/CT scans and serum tumor markers but also pathological findings, to avoid unnecessary treatment such as chemotherapy, radiation, and difficult reoperation.

## Background

When physicians suspect postoperative recurrence of lung cancer owing to elevated serum tumor marker levels and computed tomography (CT) findings, fluorodeoxyglucose (FDG)-positron emission tomography (PET)/CT is performed. FDG-PET/CT is the most sensitive non-invasive imaging method for the detection of tumor metastasis and recurrence [1]. However, PET/CT sometimes reveals false-positive results owing to infectious diseases, sarcoidosis, radiation pneumonitis, and post-operative surgical conditions [2]. Herein, we report two cases of false-positive results on PET/CT scans along with elevated serum carcinoembryonic antigen (CEA) levels, mimicking local recurrence after pulmonary segmentectomy.

## Case presentation

### Case 1

**Fig. 1:**
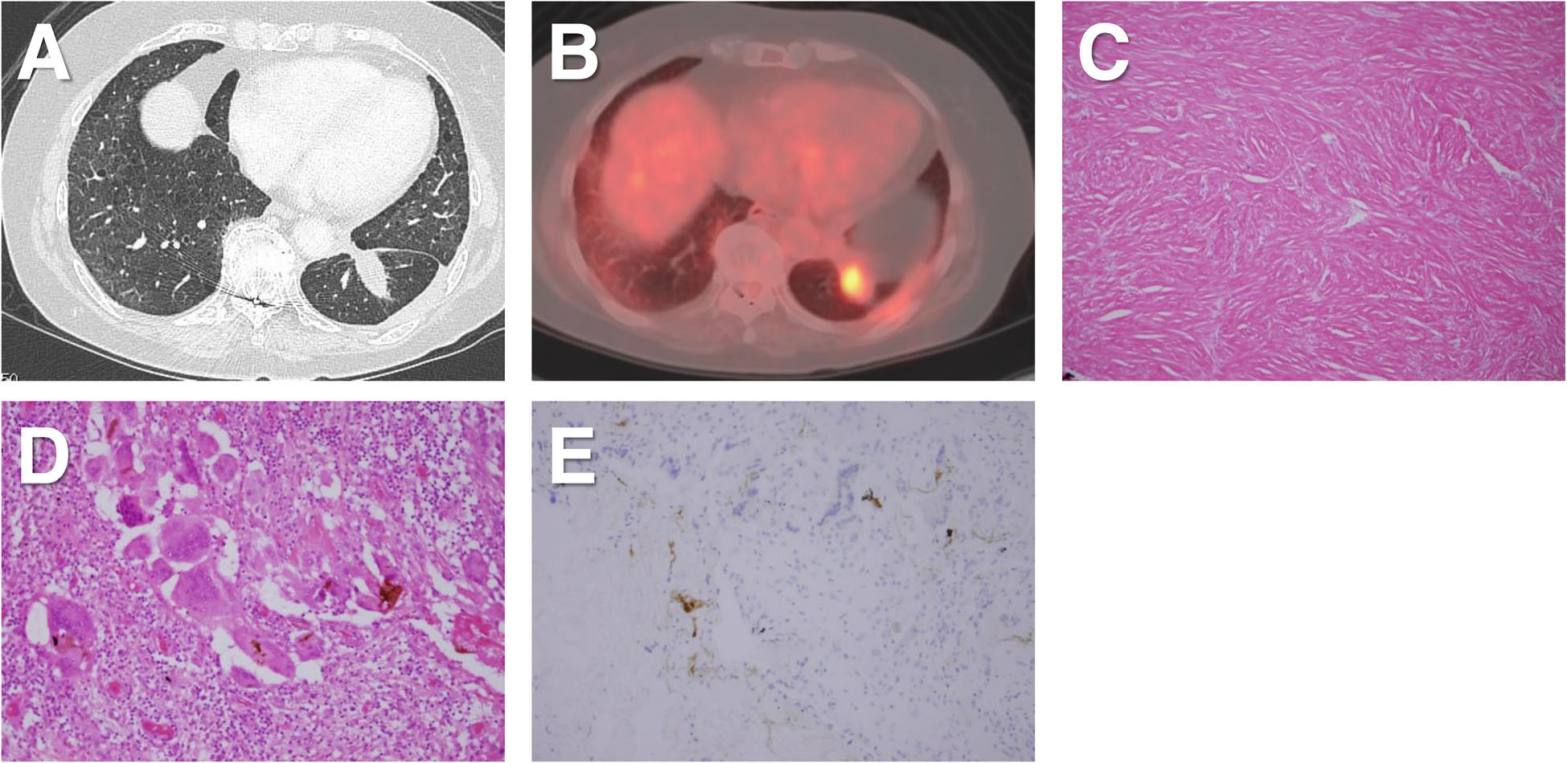
**a** Case 1: CT scan shows a nodule measuring 25 × 22 mm in the residual left lower lobe. **b** PET/CT revealed FDG uptake in the nodule (SUVmax: 5.3). **c** Microscopic view showing hyperplasia of collagen fibers (hematoxylin and eosin staining, original magnification × 100). **d** Granulomatous nodules with foreign-body giant cells (× 400). **e** Immunohistochemistry showing no CEA-positive cells in the granuloma despite CEA-positive alveolar epithelium (× 200)

A 75-year-old woman underwent video-assisted thoracic surgery (VATS) left basal segmentectomy for primary lung cancer. The intersegmental plane was cut via electrocautery, and the surface was covered with a polyglycolic acid (PGA) sheet and fibrin glue. Pathology of the resected specimen revealed a mixed adenocarcinoma (pathological stage IA). Follow-up at 6 months after the surgery revealed serum CEA level elevation to 10.0 ng/mL. Chest CT showed a nodule measuring 25 × 22 mm in the residual left lower lobe (Fig. 1a), and PET/CT revealed FDG uptake in the nodule (maximum standardized uptake value [SUVmax]: 5.3) (Fig. 1b). The pathological diagnosis of the nodule with bronchoscopic or CT guided biopsy was difficult because of the location. However, these clinical findings suggested highly suspected local recurrence of lung cancer, and the patient underwent partial resection of the nodule through thoracotomy. The total operative time was 244 min, and total blood loss was 350 mL. Pathology of the resected specimen revealed an inflammatory granuloma. There were no findings of malignancy. Microscopic examination revealed hyperplasia of collagen fibers (Fig. 1c) with granulomatous nodules and foreign body giant cells (Fig. 1d). There was no evidence of infection such as mycobacteria and fungus. On immunohistochemical examination, the granuloma and fibrous scars did not show CEA-positive cells (Fig. 1e), although CEA-positive cells were partially visible in the alveolar epithelium, and the serum CEA level decreased to 4.8 ng/mL, 7 days after the surgery.

### Case 2

**Fig. 2:**
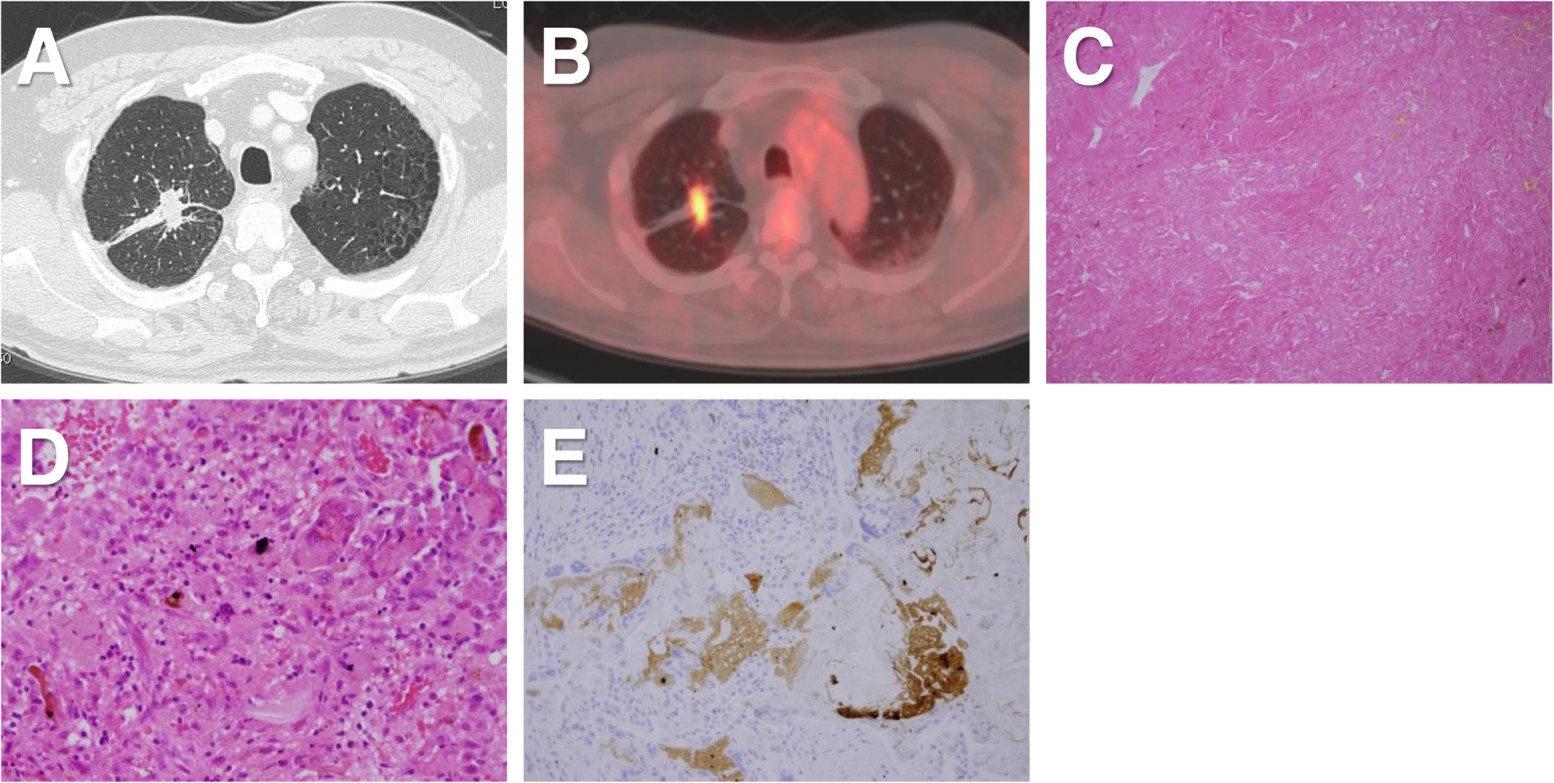
**a** Case 2: CT scan shows a nodule in the residual right upper lobe. **b** PET/CT revealed FDG uptake in the nodule (SUVmax: 6.8). **c** Microscopic view showing fibrous scars (hematoxylin and eosin staining, original magnification × 100). **d** Granulomatous nodules with foreign-body giant cells (× 400). **e** Immunohistochemistry showing no CEA-positive cells in the granuloma despite CEA-positive secretion (× 200)

A 58-year-old man underwent VATS right S1 segmentectomy for lung metastasis from rectal carcinoma. The intersegmental plane was cut via electrocautery, and the stump was suture-closed with 4–0 PGA sutures. Serum CEA levels gradually increased after surgery, and PET/CT revealed FDG uptake in the stump (SUVmax: 6.8, Fig. 2b) diagnosed as local recurrence of the lung metastasis. The patient underwent completion lobectomy through thoracotomy 6 months after the segmentectomy. The pulmonary artery tightly adhered to the surrounding tissue, and exposure of the pulmonary artery was difficult. Therefore, the total operative time was 344 min, and total blood loss was 4100 mL owing to pulmonary artery injury. Microscopy of the resected specimen revealed no malignant findings (Fig. 2c). Granulomatous nodules with foreign-body giant cells were observed in part of the fibrous scars (Fig. 2d). There was no evidence of infection such as mycobacteria and fungus. Immunohistochemistry revealed CEA positivity, although CEA-positive cells were not observed in the granuloma and fibrous scar (Fig. 2e).

## Discussion

Pulmonary granuloma is caused by inflammation; infection of bacteria, fungi, and human herpesvirus-8 [3]; and foreign bodies. Sutures and staplers also cause granuloma after lung resection [4, 5], in particular, stapler granulomas due to foreign body reaction [4, 6]. Although Munteanu et al. first reported suture granuloma by PGA suture [7], pulmonary granulomas caused by a PGA sheet have never been reported. PGA sheets are synthetic bioabsorbable meshes made with polyglycolic acid; they are useful for preventing air leakage from the lungs [8]. We usually use PGA sheets on the segmental plane cut via electrocautery during pulmonary segmentectomy. Although the mechanism by which granulomas grow is unclear, foreign body reaction may be a main cause of granuloma formation because foreign-body giant cells were observed in the granuloma. The degradation process of PGA occurs in two stages [9]. The first involves the diffusion of water into the amorphous regions of the matrix and simple hydrolytic chain scission of the ester groups. The second stage of degradation largely involves the crystalline areas of the polymer, which become predominant when the majority of the amorphous regions have been eroded. The degradation process takes only a month, but the present cases showed active granulomas at 6 months after the last surgery, suggesting that other mechanisms may have caused granuloma.

In our institution, there were six reoperations against suspected recurrence tumors between 2011 and 2017, and two of them were granulomas presented in this report. Reoperation after lobectomy and segmentectomy is occasionally performed for local recurrence of lung cancer and metastatic lung tumors. Reoperation is usually difficult and involves substantial risk because of severe adhesion surrounding the bronchial stump and pulmonary artery caused by the manipulation during initial surgery. Blood loss in Case 2 was 4100 mL, and the patient required blood transfusion. Therefore, diagnosis of local recurrence of lung malignancy is extremely important, although local recurrence usually occurs in sites difficult to perform bronchoscopic and CT-guided biopsy. PET/CT is frequently used as a diagnostic imaging modality in patients with suspected malignancy, but false-positive findings are quite common [2]. In the present cases, PET/CT revealed FDG uptake in the nodules, but both cases were false-positive findings.

Although serum CEA level measurement is useful for postoperative follow-up of malignant tumors, CEA also may show false-positive results, because the normal mucosal epithelium, such as the oral, colorectal, and bronchial mucosa, secretes CEA. Smoking and aging also result in serum CEA elevation. In the present study, both cases showed serum CEA elevation postoperatively, and CEA levels decreased after reoperation; however, the serum CEA elevation was a false-positive result in both cases. Immunohistochemistry of case 1 showed CEA-positive cells partially in the alveolar epithelium, and CEA-positive cells were observed in the granuloma site of case 2, suggesting that these cells may have elevated serum CEA levels, although it is unclear which cell type secretes CEA.

## Conclusions

We reported two cases of false-positive results on PET/CT scans and serum CEA elevation that mimicked recurrent lung cancer in the intersegmental plane after pulmonary segmentectomy. Although this appears to be the first report of granulomas caused by PGA sheets, we should consider the possibility of granulomas by sutures, staplers, and PGA sheets during the postoperative follow-up. Furthermore, recurrence of lung cancer should be diagnosed using pathological findings to avoid unnecessary treatment such as chemotherapy, radiation, and difficult reoperation.

## Data Availability

Not applicable.

## Abbreviations

CEA: Carcinoembryonic antigen
CT: Computed tomography
FDG PET/CT: Fluorodeoxyglucose-positron emission tomography / computed tomography
PGA: Polyglycolic acid
VATS: Video-assisted thoracic surgery
